# Weigh the pros and cons to ovarian reserve before stripping ovarian endometriomas prior to IVF/ICSI: A meta-analysis

**DOI:** 10.1371/journal.pone.0177426

**Published:** 2017-06-02

**Authors:** Xin Tao, Lei Chen, Shuqi Ge, Lisi Cai

**Affiliations:** 1 Center for Reproductive Medicine, the Third Affiliated Hospital of Sun-Yet Sen University, Guangzhou, Guangdong Province, China; 2 Department of Infertility and Sexual Medicine, the Third Affiliated Hospital of Sun-Yet Sen University, Guangzhou, Guangdong Province, China; Zhejiang University College of Life Sciences, CHINA

## Abstract

**Purpose:**

To explore the effects of conservative surgery for endometriomas on ovarian responsiveness during assisted reproductive technology (ART) and provide reproductive and gynecological doctors with a more reliable reference program for the treatment of endometriomas.

**Methods:**

A literature search was performed by searching the PubMed, Embase, Cochrane Library, Web of Science and Science Direct databases. Studies with inter- and intra-patient comparisons of ovarian responses and oocyte quality between operated and unoperated ovaries and that met the inclusion criteria were retrieved, and the data from the outcome measures were extracted and pooled for this meta-analysis.

**Results:**

Twenty-one published studies (2649 ART cycles) were included. The total amount of gonadotropin (Gn) used (inverse variance (IV):0.48; 95% confidence interval (CI): [0.13, 1.82], P = 0.0007) was significantly increased in the women with endometriomas who had a history of cystectomy. The estrogen (E) level on the day of hCG administration (IV: -0.29; 95% CI: [-0.41, -0.17], P<0.00001), the number of mature or dominant follicles (IV: -1.17; 95% CI: [-1.51, -0.82], P<0.00001) and the total number of oocytes retrieved (IV: -1.78; 95% CI: [-2.38, -1.17], P<0.00001) were significantly decreased in the women with endometriomas who had a history of cystectomy. The duration of stimulation (IV: 0.02; 95% CI: [-0.09, 0.13], P = 0.77), the total number of formed embryos (IV: -0.06; 95% CI: [-0.17, 0.04], P = 0.25), the pregnancy rate(IV:0.98;95%CI[0.82,1.18], P = 0.83) and the live birth rate(IV:0.93;95%CI[0.70,1.23], P = 0.61)were not statistically different between the two groups. Similar intra-patient results were found in the number of mature or dominant follicles (IV: -0.88; 95% CI: [-1.25, -0.52], P<0.00001) and the total number of oocytes retrieved (IV: -3.48; 95% CI: [-4.77, -2.19], P<0.00001).

**Conclusion:**

ART might be a better therapeutic method for ovarian endometrioma-related infertility than cystectomy.

## Introduction

Ovarian endometriomas are one of the most common benign lesions in gynecology and mainly occur in reproductive-aged women. For women with endometriomas and a strong desire to become pregnant, conservative surgery to save fertility is usually performed.

Endometriomas in infertile patients have been a clinical challenge for infertility specialists. These patients often present with infertility and are eager to achieve their reproductive needs through assisted reproductive technology (ART). The most common ART approaches are *in vitro* fertilization (IVF) and intracytoplasmic sperm injection (ICSI) with embryo transfer. However, whether ovarian surgical interventions will reduce ovarian reserves and responsiveness to stimulation is still a controversial issue. Some studies have shown that endometriomas will not damage ovarian reserves[1] and rarely damages oocyte quality[2]. Moreover, the excision of endometriomas can improve the success rate of ART[3]. However, other studies have supported the opposite view and found that endometrioma cystectomies may damage the ovarian response to controlled ovarian hyperstimulation (COH) and weaken the ovarian reserves[4–7]. Another study showed that the existence of an endometrioma affects iron metabolism in follicular fluid, increases the amount of oxygen free radicals and results in egg quality injury[8].

To better explore the effect of conservative endometrioma surgery and provide a more reliable reference program for reproductive and gynecological doctors in the treatment of endometriomas, this systematic review and meta-analysis conducted inter-patient comparisons of the outcomes of patients with or without cystectomy and intra-patient comparisons in patients with unilateral disease between the ovary that underwent the intervention and the intact ovary.

## Search strategy

The literature search was performed from January 2001 to July 2016, and related studies were identified by searching the PubMed, Embase, Cochrane Library, Web of Science and Science Direct databases. The following search terms and their combinations were used: endometrioma/endometriosis ovarian cysts, cystectomy/surgical treatment/excision, ovarian response/reserve, IVF/*in vitro* fertilization, ICSI/intracytoplasmic sperm injection, and recurrence endometrioma. The language of the publications was restricted to English. The reference lists of all retrieved studies were hand-searched to identify relevant missing studies.

## Selection criteria

The studies were included if 1) the study was a randomized controlled trial (RCT) or a retrospective comparative study (cohort and case-control studies) that compared the outcomes of endometriomas in patients with or without a conservative surgical history before IVF/ICSI and 2) had assessed the ovarian responses to COH during IVF/ICSI with at least one of the outcomes mentioned in the next section.

Grey literature, conference abstracts, letters to the editors, review articles, case reports and animal research were excluded. Endometrioma excisions mixed with other ovarian surgeries were also excluded.

If data from the same cohort were reported in more than one study, the most recent or complete study was used.

## Data extraction and outcome measures

Two investigators (Lei Chen and Xin Tao) independently extracted the data to ensure homogeneity of the data collection, and any disagreement was resolved through consultation with a third reviewer (ShuqiGe). The data collected included the study characteristics and various outcomes. The study characteristics included the first authors’ name, year of publication, study location, study design, and the details of the participants (number of group members, participant groups, intervention or COH protocol, and the location of the cysts). The following outcome measures were extracted from all included studies: total amount of gonadotropin (Gn) used (unit: IU), the duration of stimulation (days), the estrogen (E) level on the day of hCG administration (unit: pg/mL), the number of dominant follicles, the total number of oocytes retrieved, the total number of formed embryos,pregnancy rate and live birth rate. The primary measures included the results of the comparisons between the women with endometriomas with or without a conservative history of cystectomy. The secondary measures included the results of the intra-patient comparisons between the operated ovary and the unoperated ovary. When data were not available in the article, repeated efforts were made to contact the authors. Continuous data were extracted in the form of the mean±standard deviation (SD) and population sizeAnd dichotomous data were extracted in the form of the odds ratio (OR). Data were collected for all of the experienced COH cycles of every patient.

## Quality assessment

In total, 18 of the included studies were retrospective cohort and case-control studies and only 3 were RCTs. The quality of the non-randomized studies was examined in accordance with the Meta-analysis of Observational Studies in Epidemiology (MOOSE, for the observational studies) and the Newcastle-Ottawa Scale checklists[9]. The quality assessment of the RCTs was based on the Cochrane Collaboration’s tool for assessing risk of bias[10]. All differences were resolved by a consensus.

## Statistical analysis

All the meta-analyses were carried out using Review Manage 5.3 (Cochrane Collaboration, Oxford, UK), and all data retrieved were continuous variables that were analyzed using the weighted mean difference (WMD) or odds ratio(OR) with the 95% confidence interval (CI). If there was no standard deviation (SD) provided by the study, the SD was calculated using methods described by Hozo et al.[11]. Heterogeneity was evaluated graphically using forest plots and quantified using the I^2^ statistic. An I^2^>50% was represented substantial significant heterogeneity between studies. The random-effects model was used if there was significant heterogeneity between studies; otherwise, the fixed-effects model was used[12]. We screened for potential publication bias using funnel plots.

## Results

### Literature search

**Fig 1:**
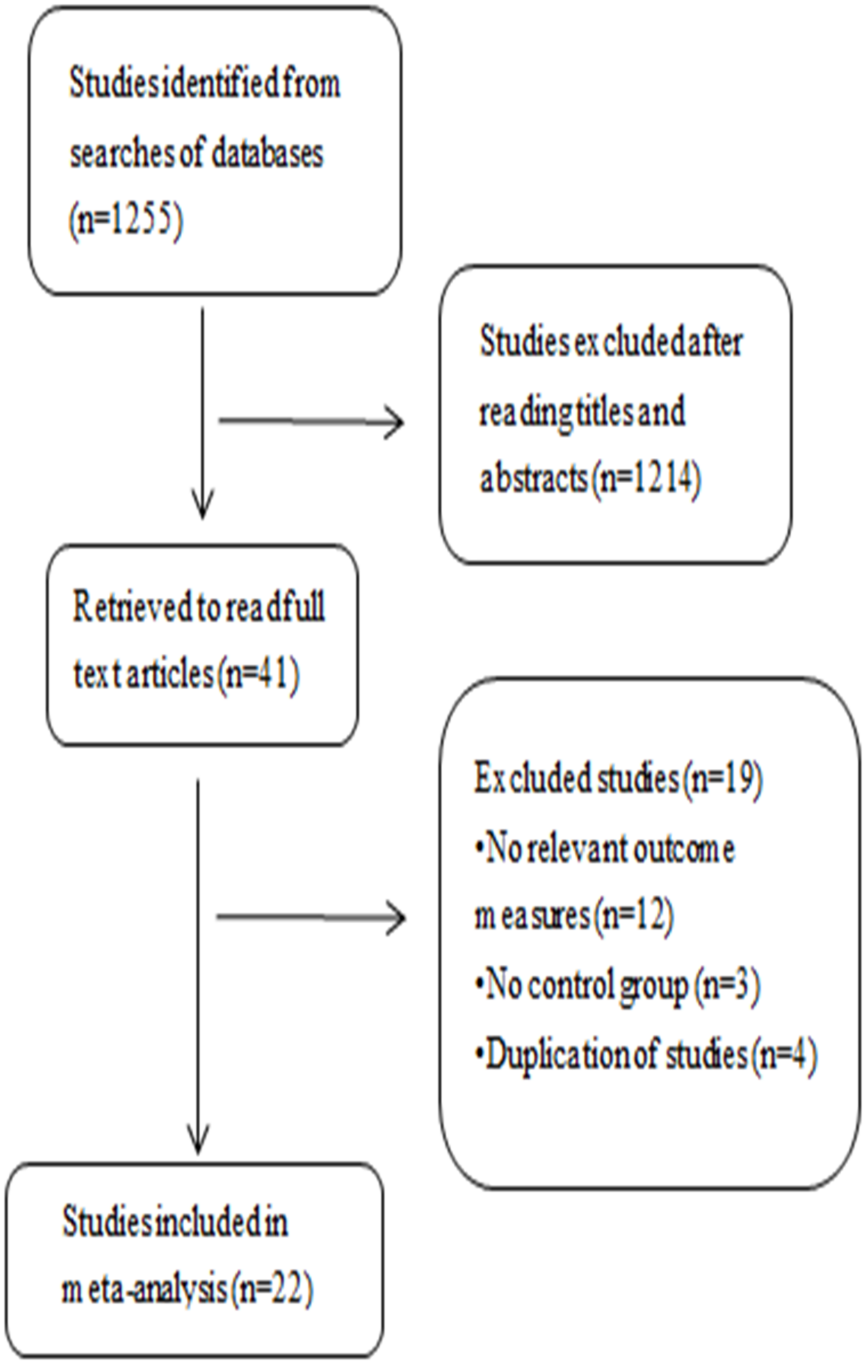
Flowchart showing the study selection process.

The literature search revealed 1255 studies from five databases (Fig 1). Of these, 1214 were excluded based on the title and abstract, and 20 were excluded after a detailed review. Therefore, 21 studies fulfilled the predefined inclusion criteria and were finally included in this meta-analysis (Fig 1) [13–34].

### Study characteristics

The characteristics of the included studies are shown in Table 1. Of the 21 included studies, 11 were conducted in Europe, 6 in Asia, 2 in North America, and 2 in South America. The number of studied cycles was 2649. The majority of the cases included were undergoing a long GnRH-agonist protocol for COH stimulation; howeverandthree studies[23,24,33] used a mixed protocol. The publication date ranged from December 2001 to July 2014. Nineteen of the retrieved studies were designed as retrospective cohort or case-control studies, and only 3 were RCTs. The 18 retrospective studies scored well on the Newcastle-Ottawa Scale, as shown in Table 1. The results of the quality assessment of the RCTs are shown in Table 2. There were 18 studies with inter-patient comparisons of the outcomes and 4 of the included studies contained intra-patient comparisons of the outcomes. All of the studies were age-matched. All of the included cases had a mean age of less than 40 years.

**Table 1:** Characteristics of all studies included in the meta-analysis.

No.	Study (authors,year)	Design	intervention/protocol	study group(N)	operated side	control group(N)	Outcome measures	Quality score	Location (country)
1	Canis et al. (2001)	Retrospective case-control	IVF long	Endometrioma cystectomy(39)	Either	surgically treated endometriosis but without endometrioma (128)	NOR	8	France
2	Geber et al. (2002)	Retrospective cohort	IVF/ICSI long	Endometrioma cystectomy(45)	Either	without ovarian surgery or endometriosis (48)	DOS,NOR,TFE,PR	8	Brazil
3	Ho. et al. (2002)	Retrospective case-control	IVF long	operated side(38)	UL	intact side(38)	NDF,NOR	8	Taiwan
4	Marconi et al. (2002)	Retrospective cohort	IVF long	Endometrioma cystectomy(39)	Either	tubal factor (39)	NOR,TFE, NDF,E2L,PR	8	Argentina
5	Pabuccu et al. (2007)	Randomize control trail	IVF/ICSI long	Endometrioma cystectomy(41)	Either	untreated endometrioma (33)	DOS,NDF, NOR,E2L,TFE, PR	RCT	Turkey
6	Pabuccu et al. (2004)	Randomize control trail	IVF/ICSI long	Endometrioma cystectomy(44)	Either	tubal factor(46)	DOS,E2L,NDFPR	RCT	Turkey
7	Wong et al. (2004)	Retrospective cohort	IVF/ICSI long	Endometrioma cystectomy(36)	Either	untreated endometrioma (38)	E2L,TFE, PR	8	USA
8	Garcia-velasco et al.(2004)	Retrospective case-control	IVF/ICSI long	Endometrioma cystectomy(147)	Either	untreated endometrioma (63)	TOG,DOS, E2L,NOR,TFE, PR	8	Spain
9	Loo et al. (2005)	Retrospective case-control	IVF ND	Endometrioma cystectomy(127)	Either	tubal factor(95)	TOG,E2L,NOR	8	Taiwan
10	Ragni et al. (2005)	Retrospective case-control	IVF/ICSI long	operated side(17)	UL	intact side(17)	NOR,NDF,	8	Italy
11	Dermirol et al. (2006)	Randomize control trail	ICSI long	Endometrioma cystectomy(49)	Either	untreated endometrioma (50)	TOG,DOS, E2L,TFE, PR	RCT	Turkey
12	Esinler et al. (2006)	Retrospective case-control	ICSI long	Endometrioma cystectomy(23)	BL	tubal factor(99)	TOG,DOS, E2L,TFE, PR	8	Turkey
13	Matalliotakis et al.(2007)	Retrospective case-control	IVF/ICSI long	Endometrioma cystectomy(133)	Either	tubal factor(208)	NDF,DOS, NOR,E2L,TFE, PR,LBR	8	USA
14	Nakagawa et al. (2007)	Retrospective cohort	IVF long	Endometrioma cystectomy(10)	Either	no endometrioma (70)	NOR,E2L,TFE PR	8	Japan
15	Somiglianaet al. (2008)	Retrospective cohort	IVF/ICSI long	Endometrioma cystectomy(68)	BL	other factor of infertility(136)	DOS,NDF, NOR,TFE,LBR	8	Italy
16	Kahyaoglu et al. (2008)	Retrospective case-control	IVF long	Endometrioma cystectomy(22)	Either	tubal factor(22)	NDF,NOR,E2L PR	8	Turkey
17	Yamamoto et al. (2010)	Retrospective case-control	IVF/ICSI long/short	Endometrioma cystectomy(35)	BL	cyst-free(94)	TOG,NOR, PR	9	Japan
18	Bongioanniet al. (2011)	Retrospective case-control	IVF long	Endometrioma cystectomy(112)	Either	untreated endometrioma (142)	TOG,NOR,TFE PR,LBR	8	Italy
19	Dong et al. (2014)	Retrospective Cohort	IVF/ICSI:long/antagonist/prolonged	Endometrioma cystectomy(153)	Either	untreated endometrioma (68)	TOG,DOS,NDFNOR,E2L,TFE, PR,LBR	8	China
20	Lee et al. (2014)	Retrospective cohort	IVF/ICSI:long/antagonist/prolonged	Endometrioma cystectomy(36)	Either	untreated endometrioma (36)	TOG,DOS,NDF,NOR,TFE, PR,LBR	8	Korea
21	Somiglianaet al. (2003)	Retrospective case-control	IVF/ICSI long	operated side(20)	UL	intact side(20)	NDF	8	Italy

Note:UL = unilateral; BL = bilateral; TOG = total of GN used; DOS = duration of stimulation; E2L = E2 level on HCG day; NDF = number of dominant follicle; NOR = number of oocyte retrieved; TFE = total formed embryos; PR = Pregnancy rate; LBR = Live birth rate; ND = not documented

**Table 2:** Risk of bias using Cochrane risk assessment tool for RCT.

Bias	Selection		Performance	Attrition	Outcome assessment	Reporting
Authors(year)	Random sequence generation	allocation sequence concealment	blinding	incomplete outcome data	blinding	selective reporting
Pabuccu et al.(2004)	low risk	low risk	high risk	low risk	low risk	high risk
Demirol et al.(2006)	low risk	low risk	high risk	low risk	low risk	high risk
Pabuccu et al.(2007)	low risk	low risk	low risk	low risk	low risk	high risk

Among the 21 selected studies, the reported outcome measures were as follows: (i) inter-patient studies: 8 reported the total amount of Gn used, 10 reported the duration of stimulation, 11 reported the E level on the day of hCG administration,10 reported the number of mature or dominant follicles, 16 reported the total number of oocytes retrieved, 13 reported the total number of formed embryos,17 reported pregnancy rate and 5 report live birth rate; and (ii) intra-patient studies: 3 reported the number of mature or dominant follicles and 2 reported the total number of oocytes retrieved.

### Primary outcomes

The primary outcomes included comparisons of the ovarian responses and oocyte quality between the cystectomy group and the control group.

#### TOG: Total amount of Gn used

**Fig 2:**
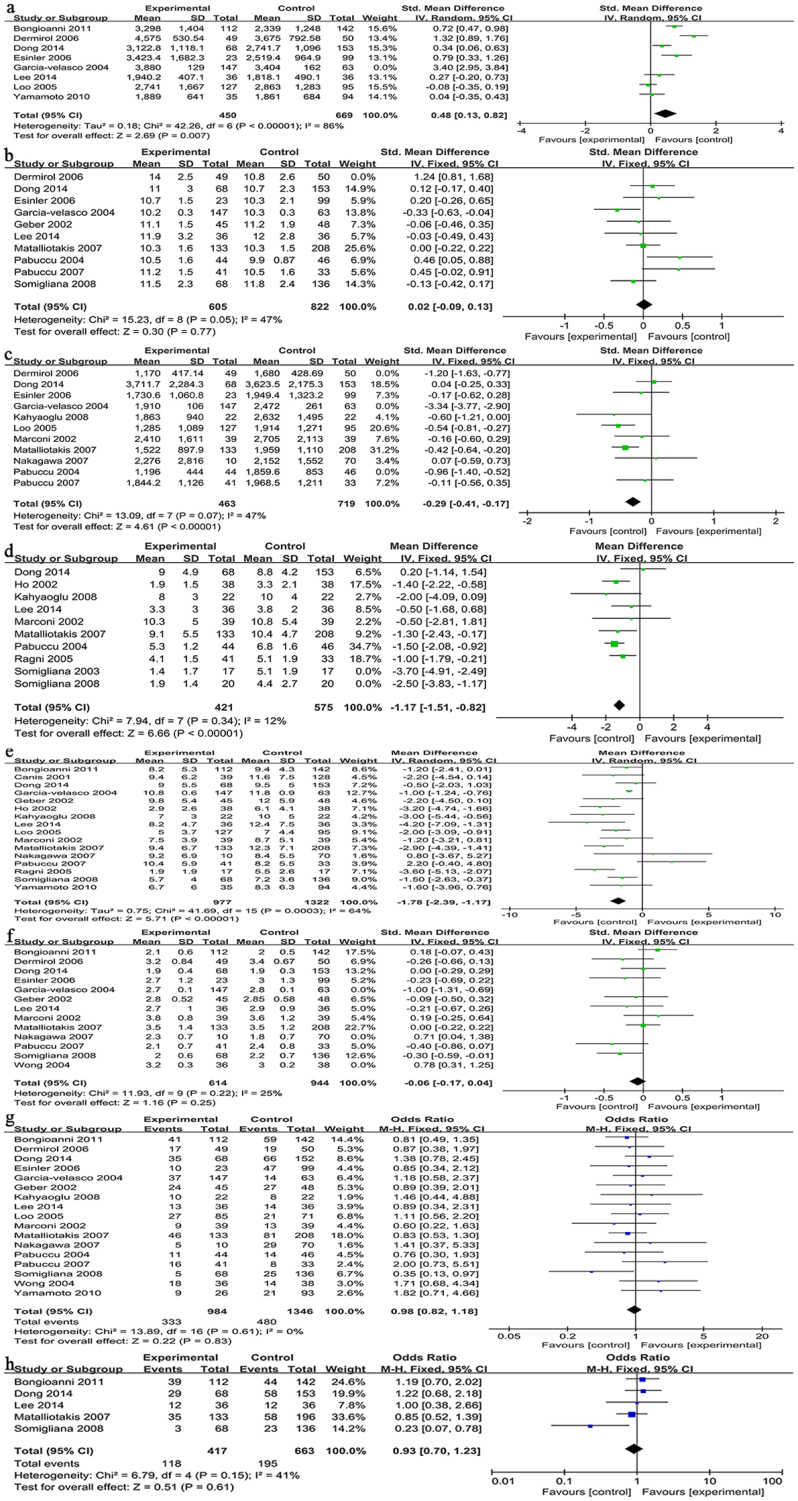
Forrest plot of comparisons inter-patient. IV = inverse variance method; CI = confidence interval; M-H = mantel-Haenszel. **(**a) Total amount of gonadotropin (Gn) used. (b) Duration of stimulation. (c) Estrogen (E) level on HCG day. (d) Number of mature or dominant follicle. (e) Total number of oocytes retrieved. (f) Total formed embryos. (g) Pregnancy rate. (h) Live birth rate.

Of the 8 studies[17–20,22–24,33] comparing the TOG between the cystectomy group and the control group, only 1[23] favored the cystectomy group, whereas 4[17–20] favored the control group and 3 showed no statistical significance. The pooled data from these 8 studies assessing the TOG in 1329 patients showed a statistically significant difference. The meta-analysis demonstrated that the TOG after cystectomy was statistically increased (inverse variance (IV): 0.84; 95% CI: [0.17, 1.51], P = 0.01) (Table 3). These 8 studies showed significant variation as indicated by an I^2^ value of 97% (P<0.0001). To analyze the source of heterogeneity, we excluded 1 study[17,19]with the highest contribution to the heterogeneity in the sensitivity analysis, and the final result was still the same (IV: 0.48; 95% CI: [0.13, 0.82], P = 0.007) (Table 4) with an I^2^ value that was slightly reduce (I^2^ = 86%). Thus,we excluded this study (Fig 2a).

**Table 3:** Results of meta-analysis comparison of cystectomy and existence of ovarian endomentrioma.

Outcomes of interest	studies no.	cystectomy patients no.	existence patients no.	WMD/95%CI	P value	heterogeneity			
						X^2^	df	I%	p value
**Primary outcomes**									
total amount of Gn used	8	597	732	0.84[0.17,1.51]	0.01	203.98	7	97%	<0.00001
duration of stimulation	10	654	872	0.17 [-0.07, 0.42]	0.17	44.39	9	80%	<0.00001
E_2_ level on HCG day	11	703	878	-0.67[-1.18,-0.16]	<0.00001	198.27	10	95%	<0.00001
number of dominant follicle	10	458	612	-1.43[-2.03,-0.84]	<0.00001	26.16	9	66%	0.002
number of oocytes retrieved	16	977	1322	-1.78[-2.39,-1.17]	<0.00001	41.69	15	64%	0.0003
total formed embryos	13	807	1115	-0.07[-030,016]	0.53	63.31	12	81%	<0.00001
pregnancy rate	17	984	1346	0.98[0.82,1.18]	0.83	13.89	16	0%	0.61
Live birth rate	5	417	633	0.93[0.70,1.23]	0.61	6.79	4	41%	0.15
**Secondary outcomes**									
number of dominant follicle	3	81	81	-1.17[-1.79,-0.56]	0.0002	6.22	2	68%	0.04
number of oocytes retrieved	2	49	49	-3.48[-4.77,-2.19]	<0.00001	0.08	1	0%	0.78

**Table 4:** Sensitivity analysis comparison of cystectomy and existence of ovarian endomentrioma.

Outcomes of interest	Studies no.	Cystectomy patients no.	Existence patients no.	WMD 95%CI	P value	heterogeneity			
						X_2_	df	I%	p value
**Primary outcomes**									
total amount of Gn used	7	450	669	0.48[0.13,0.82]	0.007	42.26	6	86%	<0.00001
duration of stimulation	9	605	822	0.02 [-0.09, 0.13]	0.77	15.23	8	47%	0.05
E_2_ level on HCG day	8	463	719	-0.29[-0.41,-0.17]	<0.00001	13.09	7	47%	0.07
number of dominant follicle	8	421	575	-1.17[-1.51,-0.82]	<0.00001	7.94	7	12%	0.34
number of oocytes retrieved	13	858	1066	-2.10[-2.73,-1.47]	<0.00001	33.32	12	64%	0.0009
total formed embryos	10	614	944	-0.06[-0.17,0.04]	0.25	11.93	9	25%	0.22
pregnancy rate	17	984	1346	0.98[0.82,1.18]	0.83	13.89	16	0%	0.61
Live birth rate	5	417	633	0.93[0.70,1.23]	0.61	6.79	4	41%	0.15
**Secondary outcomes**									
number of dominant follicle	2	64	64	-0.88[-1.25,-0.52]	<0.00001	0.46	1	0%	0.50
number of oocytes retrieved	2	49	49	-3.48[-4.77,-2.19]	<0.00001	0.08	1	0%	0.78

#### DOS: Duration of stimulation

Of the 10 studies[14,17,19,20,23–25,27–29] comparing the DOS between the cystectomy group and the control group, only 1[17] favored the cystectomy group, whereas 1[20] favored the control group, and 8 showed no statistical significance. The pooled data from these 10 studies included 1526 patients who showed no statistically significant difference in the DOS between the two groups (IV: 0.17; 95% CI: [-0.07, 0.42], P = 0.17) (Table 3). We also observed significant heterogeneity (I^2^ = 80%). In the sensitivity analysis, we excluded one study[19], and the result was still not statistically significant (IV: 0.02; 95% CI: [-0.09, 0.13], P = 0.74) (Table 4) but had lower heterogeneity (I^2^ = 47%). Thus, 1 study[19] was excluded that contributed to the heterogeneity but had no influence on the results (Fig 2b).

#### E2L: Estrogen (E) level on the day of hCG administration

Of the 11 studies[16–21,23,25,27,28,32] comparing the E2 levels between the cystectomy group and the control group, 5[17–19,25,27] favored the cystectomy group, whereas 6[16,20,21,23,28,32] showed no statistical significance. The pooled results from these 11 studies included 1581 patients and found that the E2L was significantly decreased after endometrioma stripping (IV: -0.67; 95% CI: [-1.18, -0.16], P = <0.00001) (Table 3). The I^2^ value was 95%, indicating significant heterogeneity between the studies; therefore, we then excluded 3 studies[19,20,25] in the sensitivity analysis. The result was still statistically significant (IV: -0.29; 95% CI: [-0.41, -0.17], P<0.00001) (Table 4) but with lower heterogeneity (I^2^ = 47%). Thus, these 3 studies were excluded (Fig 2c).

#### NDF: Number of mature or dominant follicles

Of the 10 studies[15,16,21,23–27,29,30] comparing the NDF between the cystectomy group and the control group, 6[15,25–27,29,30] favored the control group, whereas 4[16,21,24,25] showed no statistical significance. The pooled results from these 10 studies reporting the NDF included 1070 patients and showed a significant difference favoring the control group (IV: -1.43; 95% CI: [-2.03, -0.84], P<0.00001) (Table 3). Significant heterogeneity was observed (I^2^ = 66%); therefore, we excluded 2 studies[13, 30, 31] with the highest contribution to the heterogeneity in the sensitivity analysis, and the result was still statistically significant (IV: -1.17; 95% CI: [-1.51, -0.82], P<0.00001) (Table 4) but with much lower heterogeneity (I^2^ = 12%). Thus, we excluded these 2 studies (Fig 2d).

#### NOR: Total number of oocytes retrieved

Of the 16 studies[13–18,21–24,26–29,32,33] comparing the NOR between the cystectomy group and the control group, 8[15,17,18,21, 24,26,27,29] favored the control group, whereas 8[13,14,16,22,23,27,32,33] showed no statistical significance. The pooled data from these 16 studies included 2299 patients and showed that significantly fewer oocytes were retrieved in the cystectomy group (IV: -1.78; 95% CI: [-2.39, -1.17], P<0.00001) (Table 3). These 17 studies showed significant variation as indicated by an I^2^ value of 64% (P = 0.0003). We excluded 3 studies[24,26,28] in the sensitivity analysis, and the final result remained statistically significant (IV: -2.10; 95% CI: [-2.73, -1.47], P<0.00001) (Table 4), but the I^2^ value also remained high (I^2^ = 64%). Thus, these 3 studies were not excluded (Fig 2e).

#### TFE: Total number of formed embryos

Of the 13 studies[14,16,17,19,20,22–24,27–30,32] comparing the TFE between the cystectomy group and the control group, 2[29,32] favored the cystectomy group, whereas 2[17,31] favored the control group and 9 showed no statistical significance. The pooled data of 1922 patients in these 13 studies showed no statistically significant difference in the DOS between the two groups (IV: -0.07; 95% CI: [-0.30, 0.16], P = 0.53) (Table 3). The I^2^ value was 81% (P<0.00001), indicating significant heterogeneity between the studies; therefore, we excluded 3 studies[17,31,32] in the sensitivity analysis. The result was still not statistically significant (IV: -0.06; 95% CI: [-0.17, 0.04], P = 0.25) (Table 4) but had lower heterogeneity (I^2^ = 25%). Thus, these studies were excluded (Fig 2f).

#### PR:Pregnancy rate

Of the 17 studies[14,16–21,23–26,28–30,32–34] comparing the PR between the cystectomy group and the control group,almost all of them showed no statistical significance except 1[29] favored control group. The pooled data of 2330 patients in these 17 studies showed no statistically significant difference in the PR between the two groups (IV: 0.98; 95% CI: [1.12, 1.18], P = 0.83) (Table 3). The I^2^ value was 0% (P = 0.61), indicating that there was no evidence of heterogeneity between studies (Fig 2g).

#### LBR:Live birth rate

Of the 5 studies[22–24,27,29] comparing the PR between the cystectomy group and the control group,almost all of them showed no statistical significance except 1[29] favored control group. The pooled data of 1080 patients in these 5 studies showed no statistically significant difference in the PR between the two groups (IV: 0.93; 95% CI: [0.70, 1.23], P = 0.61) (Table 3). The I^2^ value was 41% (P = 0.61), indicating that there was a relatively low heterogeneity between studies (Fig 2h).

### Secondary outcomes

In terms of the secondary outcomes, we analyzed the intra-patient comparisons of the ovarian response and oocyte quality between the operated ovary and the unoperated ovary in the retrieved studies.

#### NDF: Number of mature or dominant follicles

**Fig 3:**
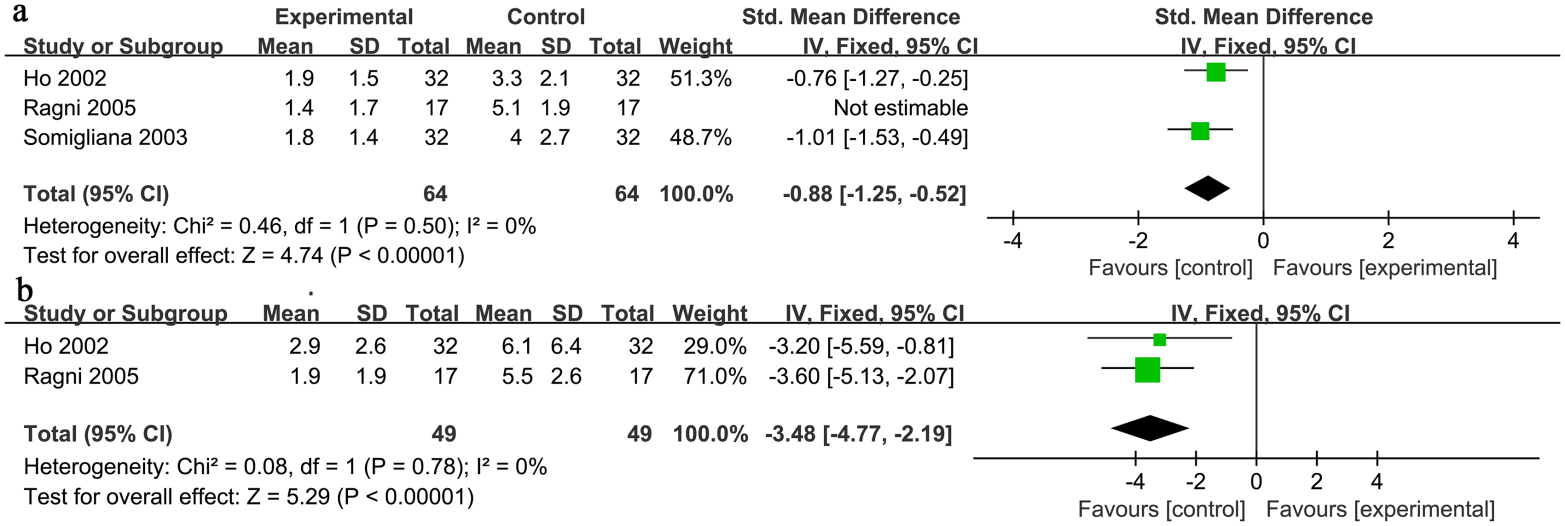
Forrest plot of comparisons inta-patient. IV = inverse variance method; CI = confidence interval. a: Number of mature or dominant follicle. b: Total number of oocytes retrieved.

Of the 3 studies[15,26,30] with intra-patient comparisons of the NDF between the operated ovary and the unoperated ovary, all of them showed that the unoperated ovary generated more dominant follicles. Similarly, the pooled data included 81 patients and statistically significantly favored the ovary without cystectomy (IV: -1.17; 95% CI: [-1.79, -0.56], P = 0.0002) (Table 3). We observed significant heterogeneity (I^2^ = 68%). In the sensitivity analysis, we excluded one study[26], and the result was still statistically significant (IV: -0.88; 95% CI: [-1.25, -0.52], P<0.00001) (Table 4) with a much lower I^2^ value (I^2^ = 0%). Thus, this study was excluded (Fig 3a).

#### NOR:Total number of oocytes retrieved

Of the 2 studies[15,26] with intra-patient comparisons of the NOR between the operated ovary and the unoperated ovary, all of them showed that less follicles were retrieved from the operated ovary. Similarly, the pooled data included 49 patients and statistically significantly favored the ovary without surgery (IV: -3.48; 95% CI: [-4.77, -2.19], P<0.00001) (Table 3). In addition, there was no evidence of heterogeneity (I^2^ = 0%) (Fig 3b).

### Sensitivity analysis and publication bias

All of the retrieved studies were included in the sensitivity analysis (Table 3). After eliminating those studies that significantly deviated from the overall mean difference, we found that the degree of between-study heterogeneity decreased significantly for the DOS, NDF, E2L and TFE inter-patien as well as the NDF intra-patient, but only a slight reduction in the heterogeneity for the TOG and NOR inter-patient were observed. Sensitivity didn’t perform in outcome mesures of PR and LBR inter-patient as well as the NOR intra-patient, as these outcome measures after pooling data were with a relatively low heterogeneity (I^2^<50%)between studies. The between-study heterogeneity remained statistically significant for all outcomes measured.

**Fig 4:**
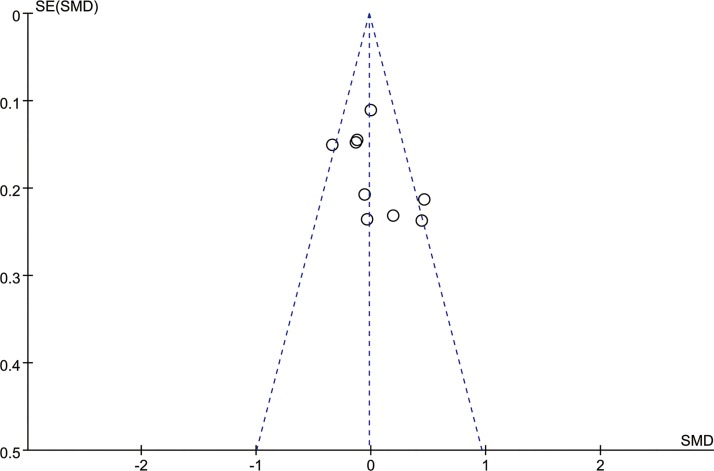
Funnel plots illustrating pregnancy rate inter-patients. SE = standard error; SMD = standard mean difference.

Fig 4 shows a funnel plot of the studies included in this meta-analysis that reported the pregnancy rate between the women with endometriomas with or without a history of conservative cystectomy. All of the studies were within the 95% CI, with an even distribution around the vertical, which indicated no obvious publication bias.

## Discussion

### Statement of the main findings

This meta-analysis of 3 RCTs and 18 retrospective studies showed that cystectomy for endometriomas reduced the number of dominant oocytes and oocytes retrieved during COH. Both the inter- and intra-patient results were similar. Moreover, endometrioma stripping increased the amount of Gn used reduced Estrogen (E) level on HCG day, in addition, cystectomy led to a reduction in Number of dominant follicle and Total number of oocytes retrieved inter- and intra-patient. However, the results indicated that there were no significant differences in the duration of stimulation, the total number of formed embryos,the pregnancy rate or the live birth rate between the two groups.

### Strengths and limitations of the study

Whether an endometrioma should be removed before IVF remains a matter of much debate. However, the results of most published studies are reported with small sample sizes. This meta-analysis included 21 studies with 2649 cycles, which made the pooled results more reliable. We conducted both inter- and intra-patient comparisons of the effect of endometriomas on cystectomy, and the intra-patient pooled data showed a reduced effect of the potential differences between patients such as body max index (BMI), sperm quality and laboratory conditions. All of the included studies scored well on the Newcastle-Ottawa Scale, suggesting a low risk of bias.

Higgins[34] reported that statistical heterogeneity is inevitable because clinical and methodological diversity always occurs in meta-analyses. Heterogeneity is calculated using the I^2^ value, and some of the I^2^ values in this meta-analysis were high, which may be explained by the small sample sizes of some of the included studies. Patient’s age and the size of the endometrioma are important for maintaining ovarian reserves during stripping. Although all included patients were younger than 40 years old and most of the diameters of the stripped endometriomas were greater than 3 cm, there were 7 studies that did not mention the size of the endometriomas. In addition, the majority of the included studies were retrospective studies and did not utilize blinding, which augments the selection bias. Due to the selection criteria for inclusion such as the language and databases accessed, we did not retrieve all possibly relevant studies. Additionally, the outcome measures in our meta-analysis were limited. There are other more reliable markers of ovarian function such as the serum level of anti-mullerian hormone(AMH), FSH/LH value, and antral follicle count (AFC). All of these measures combined could provide more reliable conclusions and interpretations of the results. Most of the included studies did not distinguish between unilateral or bilateral cystectomies, and data from the unilateral cystectomies may be not significant because the intact ovary could compensate for the deficits in ovarian reserves. Furthermore, the COH protocols were slightly different among the studies, and the surgical techniques (laparotomy or laparoscopic surgery) were not provided by all included studies. These limitations were also sources of heterogeneity.

Although we conducted a sensitivity analysis, the source of heterogeneity is still not completely clear. Owing to the limited data, we could not perform subgroup analyses. In summary, heterogeneity is difficult to avoid. Thus, the findings of this meta-analysis must be interpreted with caution.

### Implications of the study

Several recent studies have shown that patients who underwent a cystectomy had a statistically significant reduction in their ovarian reserve and responsiveness during ART[5–7]. Several possible reasons for this phenomenon can be summarized as follows: 1) a consistent amount of healthy cortical tissue is unintentionally removed together with the capsule of the cyst; 2) electrosurgical coagulation during hemostasis could play an important role in terms of damage to ovarian vascularization; and 3) a higher risk of post-operation premature ovarian failure (POF) and higher cancellation rates in IVF cycles for a reduced response of the ovaries to gonadotropins[35,36]. However, the removal of an ovarian endometrioma may provide benefits such as reducing the tension of the ovarian tissue to allow for better space for follicular development and reducing the level of inflammatory factors in the follicular fluid to improve the quality of the oocytes[8,37]. Our meta-analysis confirmed a significant decrease in the NDF and NOR in the cystectomy group.

Although our results show that endometrioma removal surgery did not have much impact on pregnancy rate and live birth rate of ART, we found that surgery increased the amount of Gn used. The existing studies have not reported that high doses of Gn used will cause any adverse effects on endometrioma, such as aggravating dysmenorrhea, diameter expansion or malignance. Even so, after endometrioma removal, cases of canceling cycle due to follicular dysplasia and ovarian hyporesponsiveness, or had difficuity in getting more high-quality folliculars are not rare, these patients often need to undergo multiple COH treatment cycles with a greatly increased cumulative treatment time and Gn usage, which is undoubtedly caused great pressure to patients in the physical, psychological and economic level. Interestingly, for the same patient, a reduction in ovarian reserves and responsiveness were also found in the cystectomized ovary compared with those in the intact ovary. This finding might be due to accidental removal of the healthy ovary’s cortex and injury to the ovarian blood supply.

We know that AMH is an indicator of earlier, more stable to response to ovarian reserve in circulation [38]. Study have found that postoperative AMH decreased significantly [39]. AFC is a more intuitive indicator to reflect ovarian reserve, study have found that AFC will not significantly decrease after endometrioma removal surgery [40]. However, more studies have shown a significant decrease in AFC after cystectomy [35,41]. In fact, in the process of endometrioma removal surgery, no matter how experienced doctors were, the normal ovarian cortex will be stripped more or less. In addition, coagulation is still the main approach for hemostasis during laparoscopic surgery nowadays, which will certainly damage the ovarian blood supply to some extent [42]. It is well known that the recurrence rate of endometriosis is very high. One study[43] showed that the recurrence rate of endometriosis after conservative surgery could reach up to 16.9%. Muzii et al.[44] found that excisional surgery for recurrent endometriomas appears to be more harmful to the ovarian reserve, as evaluated by AFC.

At present, laparoscopic excision is still a very common practice for the surgical treatment of ovarian endometriomas, particularly in cases associated with pain and large diameter endometriomas. The European Society of Human Reproduction and Embryology (ESHRE) guidelines recommend laparoscopic ovarian cystectomy if the endometrioma is ≥3 cm in diameter in order to confirm the diagnosis histologically and possibly improve ovarian responsiveness[45]. The pelvic microenvironment is essential for reaching individual treatment goals (short-term and long-term fertility goals) for infertility with the least adverse effects on the healthy ovarian tissue. Surgeons should collaborate with specialists in reproductive medicine and determine management strategies together. Surgery for the removal of endometriomas should be performed following appropriate techniques, such as utilizing sutures instead of electrosurgical coagulation for bleeding, and by experienced surgeons in order to decrease the possibility of a post-surgical reduction in ovarian reserves.

Our meta-analysis found that cystectomy for endometriomas may diminish the ovarian reserve and reduce the responsiveness to COH. Endometriomas *per se* can cause endometrioma-related injury to the ovary. Although the removal of the endometrioma would reduce endometrioma-related injury, it may result in further surgery-related injury. Both historical and surgical factors have a gonadotoxic effect on the surrounding follicles[46], which should be considered.

In conclusion, our study provides relatively statistically powerful findings with a large number of participants. Our aim was to assess the effect of cystectomy on ovarian reserves and responsiveness to stimulation. The results indicated that cystectomy of endometriomas reduces the number of dominant oocytes and the number of oocytes retrieved during COH. Both the inter- and intra-patient results were similar. Moreover, endometrioma stripping increased the amount of Gn used. The results of this study also suggested there were no significant differences in the duration of stimulation and the total number of formed embryos between the cystectomized ovary and the intact ovary.and the pregnancy rate and live birth rate seem no statistically significant difference between the two groups.Our data indicated that ART might be a better therapeutic method for ovarian endometrioma-related infertility than cystectomy. Due to the potential publication bias and evidence of heterogeneity, further well designed, robust data trials on the management of endometriomas before ART are required.

## Supporting information

S1 Table PRISMA 2009 checklist-17. (DOC)
