# Supplementary material for: Weigh the pros and cons to ovarian reserve before stripping ovarian endometriomas prior to IVF/ICSI: A meta-analysis
Source: PLoS One. 2017 Jun 2;12(6):e0177426. doi: 10.1371/journal.pone.0177426 (PMC5456033; doi:10.1371/journal.pone.0177426)
Supplement: S1 Table — (DOC) [file pone.0177426.s001.doc]

| **Section/topic** | **#** | **Checklist item** | **Reported on page #** |
| --- | --- | --- | --- |
| **TITLE** | | |  |
| Title | 1 | **The report is identified as a meta-analysis** | Page 1. |
| **ABSTRACT** | | |  |
| Structured summary | 2 | Registration dose not apply,the background; objectives; data sources; study eligibility criteria, participants, and interventions; study appraisal and synthesis methods; results; limitations; conclusions and implications of key findings are described in the manuscript. | Page 1-2. |
| **INTRODUCTION** | | |  |
| Rationale | 3 | To explore the effect of conservative surgery of endometrioma on ovarian responsiveness during Assisted Reproductive Technology(ART), and provide a more reliable reference program for treatment of endometrioma for reproductive and gynecological doctors. | Page 2. |
| Objectives | 4 | To better explore the effect of conservative surgery of endometrioma, provide a more reliable reference program for treatment of endometrioma to reproductive and gynecological doctors. | Page 2. |
| **METHODS** | | |  |
| Protocol and registration | 5 | There is not a review protocol exists. | / |
| Eligibility criteria | 6 | Specify study characteristics and report characteristics are described in the manuscript. | Page 3. |
| Information sources | 7 | The literature search was performed from January 2001 to July 2016 and related studies were identified by searching PubMed, Embase, Cochrane Libraries, Web of Science and Science Direct. | Page 3. |
| Search | 8 | Pubmed:(((((endometrioma[Title/Abstract]) OR Endometriosis ovarian cysts[Title/Abstract])) AND (((cystectomy[Title/Abstract]) OR surgical treatment[Title/Abstract]) OR excision[Title/Abstract])) OR ((ovarian response[Title/Abstract]) OR reserve[Title/Abstract])) AND ((((IVF[Title/Abstract]) OR in vitro fertilization[Title/Abstract]) OR ICSI[Title/Abstract]) OR intracytoplasmic sperm injection[Title/Abstract]) | Hadn’t show in the manuscript. |
| Study selection | 9 | The studies were included if:1)the study was randomized controlled trails(RCTs) and retrospective comparative studies(cohort and case-control studies) that compared outcomes of endometrioma with or without conservative surgical history before IVF/ICSI; 2) had assessed ovarian response to COH during IVF/ICSI of at least one of the outcomes mentioned in the next section. | Page 3. |
| Data collection process | 10 | The characteristics we extracted from each studies included the first authors’ name,year of publication, study location,design of study,details of the participants (number of group members, group of participants, intervention or COH protocol, location of the cysts).The following outcome measures were extracted from all of the included studies:total amount of gonadotropin(Gn) used(unit:IU),duration of stimulation (days),estrogen(E) level on HCG day (unit:pg/mL), number of dominant follicle,total number of oocytes retrieved,total formed embryos. | Page 4. |
| Data items | 11 | Funding sources,any assumptions and simplifications made are described in the manuscript. | Page 14. |
| Risk of bias in individual studies | 12 | Quality assessment of Randomized control trails is based on the Cochrane Collaboration’s tool for assessing risk of bias. | Page 4-5.  Attachment:Table1and2 |
| Summary measures | 13 | Continuous data were extracted in the form of mean value±standard deviation (SD) and population size. | Page 5. |
| Synthesis of results | 14 | Heterogeneity was evaluated graphically using forest plot and quantified using the I^2^ statistic.I^2^＞50% was considered to represent substantial significant heterogeneity between studies. | Page 5. |

Page 1 of 2

| **Section/topic** | **#** | **Checklist item** | **Reported on page #** |
| --- | --- | --- | --- |
| Risk of bias across studies | 15 | Publication bias across studies with a funnel plot as attachment:figure 2. | Attachment  Figure 2 |
| Additional analyses | 16 | We conducted sensitivity analysis which were pre-specified,the source of heterogeneity is still not completely clear. Owing to the limited data,we couldn’t perform subgroup analysis. | Page 10. |
| **RESULTS** | | |  |
| Study selection | 17 | 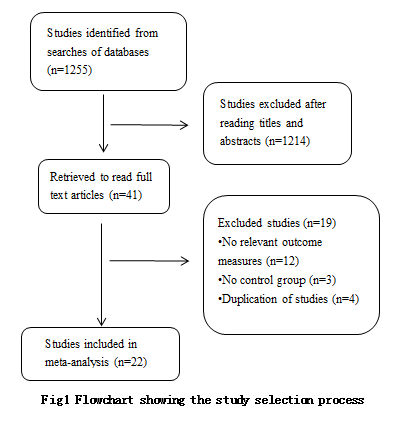 | Attachment  Figure 1 |
| Study characteristics | 18 | Characteristics of each study for which data were extracted are described as attachment: Table 1. | Attachment  Table 1. |
| Risk of bias within studies | 19 | Risk of bias within studies were show in Table1 and 2:quality score. | Page 6 and Attachment  Table 1. |
| Results of individual studies | 20 | All outcomes are presented with forest plots as attachment:figure 3 and 4. | Attachment  figure3 and 4. |
| Synthesis of results | 21 | We have presented results of each meta-analysis, including confidence intervals and measures of consistency. | / |
| Risk of bias across studies | 22 | Data on risk of bias of each study are available with a funnel plot as attachment:figure2 | Attachment  Figure2 |
| Additional analysis | 23 | Results of sensitivity analyses are available in the manuscript. | Page 3. |
| **DISCUSSION** | | |  |
| Summary of evidence | 24 | The main findings are summarized in the manuscript. | Page 11. |
| Limitations | 25 | We have discussed limitations at study and outcome level and at review-level. | Page 11. |
| Conclusions | 26 | We have provided a general interpretation of the results in the context of other evidence, and implications for future research. | Page 12. |
| **FUNDING** | | |  |
| Funding | 27 | We have described sources of funding for the systematic review and other support; role of funders for the systematic review. | Page 14. |

*From:*  Moher D, Liberati A, Tetzlaff J, Altman DG, The PRISMA Group (2009). Preferred Reporting Items for Systematic Reviews and Meta-Analyses: The PRISMA Statement. PLoS Med 6(7): e1000097. doi:10.1371/journal.pmed1000097

For more information, visit: **www.prisma-statement.org**.

Page 2 of 2
